# The Reorganisation of the Teaching of Medicine and Surgery

**Published:** 1919-09-20

**Authors:** 


### MEDICAL EDUCATION AT LONDON HOSPITALS.

### The Reorganisation of the Teaching of Medicine and Surgery.

Last year Sir George Newman published his Memorandum on Medical Education. This Memorandum covered a wide field, but considerable stress was laid on the clinical teaching of medicine and surgery, and certain recommendations were made for its improvement. The Board of Education have now taken steps to give effect to these recommendations, and four of the great London hospitals—St. Bartholomew's, St. Thomas's, the "London," and University—have been invited to submit schemes for the reconstruction and improvement of clinical teaching. Provided that these schemes meet with approval, the Board will undertake to defray three-quarters of the cost, and the medical schools will be required to pay the remaining quarter.

The lines of reconstruction are indicated in the Memorandum. Briefly, it is recommended that professors of clinical medicine, surgery and obstetrics be appointed. These professors, if not giving their whole time, are, at any rate, to give the greater part of their time to the work. They are to be adequately paid, with a sufficient number of paid assistants. Sixty to eighty beds are to be allotted to each unit, with laboratories, an out-patient department, and clerical assistance.

In the intervening time between the publication of the Memorandum and the giving effect thereto a good deal of discussion and speculation has taken place, and much uncertainty exists as to what will be the exact functions of the proposed units, and which is the trend of the recommendations. Will the result be the gradual extinction of the system of clinical teaching by a voluntary staff and the cutting-up of the teaching hospitals into numerous units, each with a paid professor, or will academic units in medicine, surgery, and obstetrics be established, distinct and self-contained, while the ordinary clinical units continue as heretofore? On the other hand, it is possible that the Board of Education contemplate not so much the uprooting of the present system, but rather its reconstruction and improvement, and much can be said in favour of this last alternative.

An independent critic has stated that the English general practitioner is the best in the world, but this does not mean that he is incapable of improvement. It must be admitted that much of the present-day clinical teaching is haphazard and unorganised. The excellence of English general practitioners is due in no small measure to the system by which students are given responsibility—whether as dressers or clerks, or, when qualified, as house physicians or surgeons, they have a certain amount of actual responsibility for patients, and no amount of demonstrations and lectures can take its place. But the fact remains that the lectures and demonstrations are capable of considerable improvement. At present they are carried out by physicians and surgeons, eminent in many cases, but encumbered with large private practices, and for whom clinical teaching is only a secondary consideration. Whereas in anatomy, physiology, etc., there are professors and demonstrators whose sole work is the teaching of their subjects. The clinical teaching of medicine and surgery, the culminating point of the student's career, is nobody's chief concern, but is left to the *horæ subsecivæ* of a staff often overworked in other ways.

Yet it is not so much the actual teaching which is at fault, but rather its lack of organisation. At most of our great hospitals there are excellent teachers of various branches of medicine and surgery, and these teach their own speciality, but independently and without any regard to the curriculum as a whole. There is no one individual whose chief work it is to map out, say, the medical clinical teaching of the student. The result is that, though there may be many excellent lectures and demonstrations in various branches of medicine and surgery for a student to attend, there is an absence of organisation of the curriculum as a whole. If on the staff of the particular hospital at which the student attends there is no neurologist, he gets no lectures in neurology, and it is no one's business to see that the deficiency is rectified.

The right conception of the professor, the head of a unit, is not merely an individual who has his own team, his own beds, his own out-patient department and laboratories, and who works isolated from his colleagues and the rest of the hospital, but rather an individual who, while devoting the greater part of his time to clinical teaching, specialising, in fact, in teaching, and raising its standard, makes use of all the resources of the hospital. One single individual cannot be a specialist in every branch of medicine, but the professor would work into a comprehensive scheme those members of the staff particularly qualified to teach special subjects, and he would see that those who cannot teach are not required to do so. He would organise the resources so that the student would have his time mapped out, and at the end of his course of clinical teaching in medicine, surgery, or obstetrics, would have received instructions from the professor and those members of the staff best qualified to teach, and would have covered as wide a field as thoroughly as possible in the time at his disposal.

### Guy's Hospital Scheme.

We understand that at Guy's Hospital a very comprehensive scheme for the reorganisation of the teaching of medicine and surgery on different lines to those indicated above has been formulated by the Medical School, and has been sanctioned by the Governors, subject to such modifications as may be introduced by the House Committee after consultation with the medical and surgical staff.

**Medical Education at London Hospitals, Guy's**—(*cont.*).

Whilst not ultimately excluding the establishment of professorial units, the first aim of those responsible for it has been to develop "team work," each "team" or "firm" being grouped around the present physicians and surgeons, whether in the general or special departments, and also to develop better co-ordination between the workers in all branches, and to bring clinical investigations into closer contact with laboratory research.

We hope in a later issue to give a fuller account of the Guy's scheme, which it will be interesting to contrast with the plans which are understood to be now before the Board of Education.

